# Malaria in Brazil, Colombia, Peru and Venezuela: current challenges in malaria control and elimination

**DOI:** 10.1186/s12936-017-1925-6

**Published:** 2017-07-04

**Authors:** Judith Recht, André M. Siqueira, Wuelton M. Monteiro, Sonia M. Herrera, Sócrates Herrera, Marcus V. G. Lacerda

**Affiliations:** 1 Independent consultant, Recife, Brazil; 2 Instituto Nacional de Infectologia Evandro Chagas, Fundação Oswaldo Cruz (Fiocruz), Rio de Janeiro, Brazil; 3 Diretoria de Ensino e Pesquisa, Fundação de Medicina Tropical Dr. Heitor Vieira Dourado, Manaus, Amazonas Brazil; 4 Centro de Investigación Científica Caucaseco, Cali, Colombia; 5 Fiocruz/Fundação de Medicina Tropical Dr. Heitor Vieira Dourado/Institute Elimina, Manaus, Brazil

**Keywords:** *Plasmodium*, Control, Elimination, Eradication, Amazon, South America

## Abstract

In spite of significant progress towards malaria control and elimination achieved in South America in the 2000s, this mosquito-transmitted tropical disease remains an important public health concern in the region. Most malaria cases in South America come from Amazon rain forest areas in northern countries, where more than half of malaria is caused by *Plasmodium vivax,* while *Plasmodium falciparum* malaria incidence has decreased in recent years. This review discusses current malaria data, policies and challenges in four South American Amazon countries: Brazil, Colombia, Peru and the Bolivarian Republic of Venezuela. Challenges to continuing efforts to further decrease malaria incidence in this region include: a significant increase in malaria cases in recent years in Venezuela, evidence of submicroscopic and asymptomatic infections, peri-urban malaria, gold mining-related malaria, malaria in pregnancy, glucose-6-phosphate dehydrogenase (G6PD) deficiency and primaquine use, and possible under-detection of *Plasmodium malariae*. Some of these challenges underscore the need to implement appropriate tools and procedures in specific regions, such as a field-compatible molecular malaria test, a *P. malariae*-specific test, malaria diagnosis and appropriate treatment as part of regular antenatal care visits, G6PD test before primaquine administration for *P. vivax* cases (with weekly primaquine regimen for G6PD deficient individuals), single low dose of primaquine for *P. falciparum* malaria in Colombia, and national and regional efforts to contain malaria spread in Venezuela urgently needed especially in mining areas. Joint efforts and commitment towards malaria control and elimination should be strategized based on examples of successful regional malaria fighting initiatives, such as PAMAFRO and RAVREDA/AMI.

## Background

Although significant advances have been made towards malaria elimination in several endemic countries in the Americas, malaria is still an important public health concern. In Latin American tropical and sub-tropical areas there are still several malaria endemic regions that impose a considerable burden on local populations. The majority of malaria cases in South America occur in the Amazon region. In 2015, the four countries reviewed here accounted for 83% of malaria cases in the Americas: Brazil (24%), Bolivarian Republic of Venezuela (30%), Colombia (10%), and Peru (19%) [1]. However, because of a significant decrease in the number of malaria cases during the last decade, several countries in South America are making progress towards eventual malaria elimination, with the notable exception of the Bolivarian Republic of Venezuela (hereinafter referred to as Venezuela) experiencing an alarming increase in malaria in recent years.

**Fig. 1:**
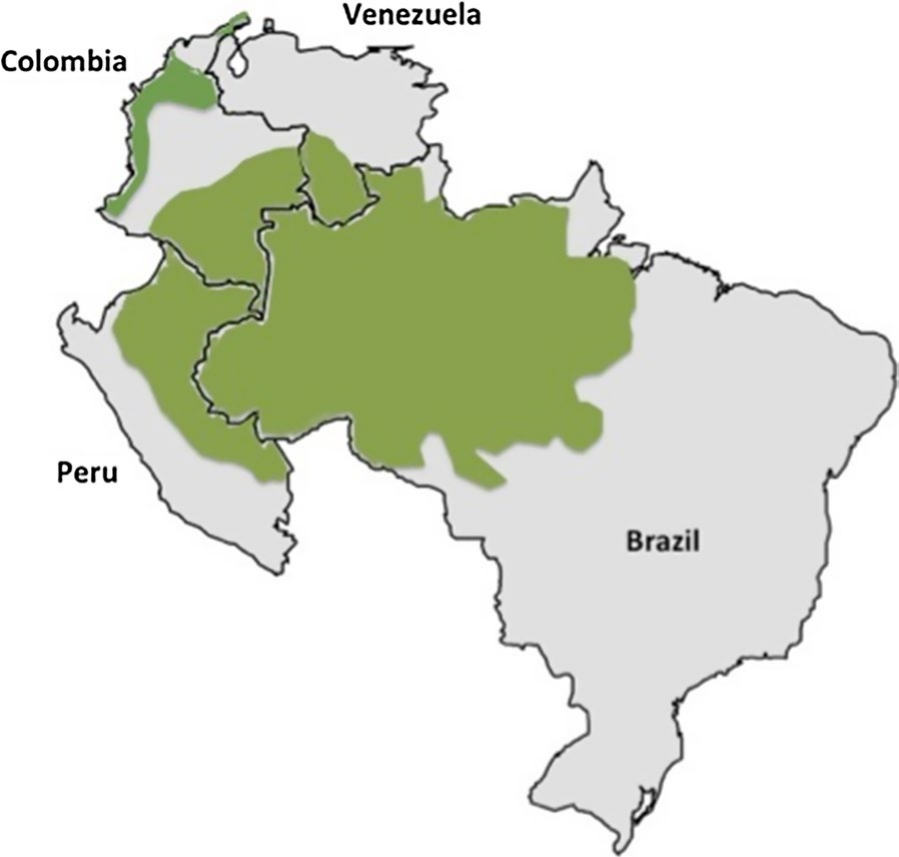
South American malaria endemic countries in this review. Brazil, Colombia, Peru and Venezuela are shown. *Green* indicates Amazon rain forest areas, where a majority of malaria cases are reported in each of the countries except in Colombia where a considerable contribution of malaria cases comes from the Pacific coast. Colombian Pacific and Caribbean coastal forest areas are also shown in *green*

Within the Americas, populations in the Amazon are the ones at highest risk of malaria infection [2]. Here four countries in South America are reviewed where malaria, with low and unstable transmission, is still endemic: Colombia, Brazil, Peru and Venezuela. All of these countries have regions in the Amazon rainforest (Fig. 1), which in the case of Peru and Brazil is where most of the malaria cases come from. Only approximately 0.5% of the malaria cases in Brazil (2000–2013) were diagnosed and treated outside the Amazonian endemic region [3]. Colombia is the exception with one of its most malaria endemic regions on the Pacific coast reporting the majority of malaria cases, populated mainly by Afro-Colombian and indigenous communities along the coastal forests (reviewed in [4]).

In South America, Argentina and Paraguay are in elimination phase with malaria elimination certified by the World Health Organization (WHO) after three consecutive years of no indigenous cases (2013–2015) [1]. Ecuador (bordering Colombia and Peru on the Pacific coast) has entered malaria pre-elimination phase, whereas Colombia, Brazil, Peru and Venezuela are still in control phase [1]. In Brazil, a *Plasmodium falciparum* elimination plan was launched a couple of years ago. This review covers the current malaria landscape of these four countries, focusing on specific challenges that need to be addressed in order to advance towards malaria elimination in this region with Amazon countries. The WHO 2016 World Malaria Report [1], the 2015 report from the Amazon malaria initiative (AMI) [5], data and alerts issued by the Pan American Health Organization (PAHO) as well as from countries’ Ministries of Health (MoHs) were sources of most recent regional and country-specific data and activities. Unpublished data from authors’ research was included where indicated.

## Recent malaria incidence trends in Brazil, Colombia, Peru and Venezuela

**Fig. 2:**
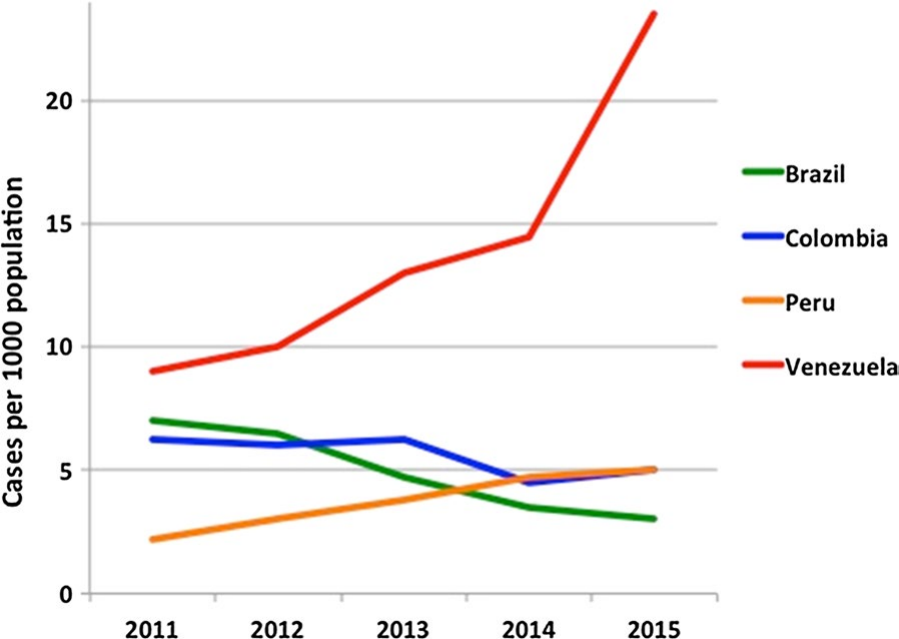
Confirmed malaria cases 2011–2015. Number of confirmed malaria cases in Brazil, Colombia, Peru and Venezuela are shown per 1000 population Data from [1]

Most countries in the Americas have shown an impressive decreasing trend in malaria incidence over the first decade of this Century/Millennium (reviewed in [6]), including all countries in this review with the exception of Venezuela. However, from the four neighbouring countries in South America reviewed here (Brazil, Colombia, Peru and the Venezuela) in recent years only Colombia and Brazil have continued to show a gradual decrease in the number of confirmed malaria cases per 1000 population, whereas Peru and Venezuela have experienced increases (Fig. 2). In the Loreto region in the Peruvian Amazon, malaria nearly tripled between 2011 and 2014, with 60,566 reported cases in 2014. Furthermore, PAHO just issued an alert in February of 2017 pointing to a recent increase in malaria cases in several countries in the Americas including both Colombia and Venezuela [7] based on data from non-WHO sources for the year 2016: 83.356 malaria cases were reported in Colombia with 57% (47,497) caused by *P. falciparum* 39.7% (33,055) by *Plasmodium vivax,* and 3.3% (2804) were mixed infections [8], whereas in Venezuela there were 240,613 cases in 2016, a 75% increase over 2015 cases for this country (136,402) [9].

## Malaria-causing parasites

Human malaria is caused worldwide by at least five different species of the genus *Plasmodium*: *P. falciparum, P. vivax, Plasmodium malariae, Plasmodium ovale* and *Plasmodium knowlesi*. In most endemic regions, malaria is due mainly to infection by either *P. falciparum* or *P. vivax*. The proportion of these two parasite species varies geographically, depending in part on susceptibility to infection of specific populations. In Africa, Duffy-negative (Fy−) blood group is widespread [10] and there is a predominance of *P. falciparum* over *P. vivax* infections. This has been attributed to the model by which *P. vivax* parasites require attachment to the Fy antigen (receptor) on erythrocytes, therefore the absence of Fy antigen results in protection from *P. vivax* malaria infection, although there are reports of vivax malaria in Duffy-negative individuals in Africa. In the Americas, including the four South American countries reviewed here, there has been an increasing relative predominance of *P. vivax* infections [1] although some regions exhibit a high prevalence of Fy− phenotypes in Afro-descendant populations.

### *Plasmodium falciparum* and *Plasmodium vivax*

In the Americas, *P. vivax* accounts for the majority of malaria cases. In the countries reviewed here there is also a majority of *P. vivax* over *P. falciparum* cases, except for some regions in Colombia where the proportion of these parasite species changes and a greater proportion of *P. falciparum* was observed overall in 2016 (Table 1). In some regions of the Colombian Pacific coast such as Chocó and Nariño departments there is a high prevalence of Fy− and consequently there is a larger proportion of *P. falciparum* cases [4]. In 2016, three departments in this region (Chocó, Nariño and Antioquia) contributed 78.1% of total malaria cases and over 87.8% of *P. falciparum* cases [8]. In Brazil, the incidence of *P. falciparum* and *P. vivax* cases was similar until about 1988, after which the proportion of *P. falciparum* malaria progressively declined while *P. vivax* increased and became the predominant species responsible for over 90% of malaria episodes in 2011 (reviewed in [11, 12]).

**Table 1:** *P. falciparum:P. vivax* malaria cases ratio in recent years

	Brazil	Colombia	Peru	Venezuela
*P. falciparum:P. vivax* (% of total malaria)^a^				
2014	18:82	34:66	16:84	35:65
2015	16:84	50:50	16:84	31:69
2016	12:88	60:40	21:79	26:74

^a^From the WHO World Malaria Reports for 2014, 2015 and 2016 [1, 47, 146]

Recent review highlights why biological differences between *P. vivax* and *P. falciparum* make *P. vivax* particularly challenging to control and eliminate in malaria endemic regions [13–15]. These reasons include lower parasite densities (about one order of magnitude lower than *P. falciparum* infections) which go undetected and, therefore, sometimes untreated resulting in higher levels of transmission to mosquitoes (enhanced by development of *P. vivax* gametocytes at earlier stages compared to *P. falciparum*, before infection is diagnosed/treated), a faster growth capacity in the mosquito vector and at lower temperatures, the presence of hypnozoites (dormant liver forms which can only be eliminated by the anti-malarial drug primaquine) that allow relapses to occur at later times, and natural immunity acquired earlier in life compared to than against *P. falciparum*. Moreover, although malaria caused by *P. vivax* was for a long time considered benign in terms of severity of disease compared to falciparum malaria, recently this perception has changed, and vivax malaria has emerged as a neglected potentially severe disease, in particular in Southeast Asia and South America [16] with an important burden on morbidity and mortality on affected populations.

In Latin America, previously neglected vivax malaria is now emerging as an important focus of research in regions where the majority of malaria infections are caused by this parasite (reviewed in [17]). In Peru, a retrospective case control study recently reported that between 2008 and 2009 in a region with nearly exclusive vivax malaria transmission, of 81 hospitalized cases classified as severe, 28 individuals were critically ill with severe anaemia (57%), shock (25%), lung injury (21%), acute renal failure (14%), or cerebral malaria (11%), and two potentially malaria-related deaths occurred [18]. Severe cases of vivax malaria have also been reported in Brazil in endemic areas in the Amazon [19–22]. In Manaus (Brazilian Amazon’s largest city) from 316 patients admitted with vivax malaria at reference tertiary hospitals between 2009 and 2011, 40 were severe vivax malaria cases; 19 of these had severe anaemia, the most common complication, followed by acute renal failure and respiratory distress, and three of these 40 patients died [21]. An interesting observation in this study was that anti-malarial treatment initiation could have been the cause of respiratory distress, for which the authors suggest further investigation.

In Colombia, studies point to higher morbidity and mortality for *P. falciparum/P. vivax* mixed infections [23, 24]. Among Embera (Amerindian) children under 14 years of age in an endemic area in Colombia in 2013, there were 22 presenting one or more criteria for severe vivax malaria (severe anaemia, renal dysfunction, respiratory distress and/or seizure) [24]. In a Colombian passive surveillance study involving 1328 patients from 2011 to 2013 with *Plasmodium* spp. infections at four malaria endemic areas of the Pacific coast only 7.5% of the cases were classified as clinically severe malaria, caused by both *P. vivax* and *P. falciparum* [25]. In an ongoing study on 326 hospitalized cases of complicated malaria due to *P. vivax* or *P. falciparum* infections at tertiary hospitals in malaria endemic areas of Colombia with special emphasis on the Pacific coast, severe thrombocytopaenia (43%), hepatic dysfunction (40%), and severe anaemia (34%) where the most common complications (M. Arevalo-Herrera, pers. commun.).

### Is *Plasmodium malariae* infection under-detected in South America?

Although not usually acknowledged because of its low incidence in Latin America (<1%), *Plasmodium malariae* is however another malaria-causing parasite. It differs from other *Plasmodium* species by exhibiting a lower growth rate and very low levels of parasitaemia. Human infection by *P. malariae* is often asymptomatic and long-lasting (up to last 40 years in the absence of re-infection) with blood stages probably persisting for a lifetime [26–29]. Patient evaluation has been reported due to recrudescence induced by splenectomy performed for malaria-unrelated reasons [30] or when malaria is acquired from transfusions with *Plasmodium*-infected blood, as observed in Latin America [31].

When this parasite causes symptoms, it results in quartan malaria or 72 h (3-day) fever cycles, as opposed to all other human malaria parasites whose infection results in 2-day (tertian fever) cycles. *P. malariae* is considered a milder pathogen compared to *P. falciparum* and *P. vivax*, except it can result in cases of chronic nephropathy. In Africa, the most commonly observed malaria corresponds to *P. malariae* and *P. falciparum.* When *P. malariae* is diagnosed as a mono-infection in Brazil, it is treated with chloroquine (25 mg/kg over 3 days); for coinfections the treatment is chloroquine with the addition of primaquine for *P. vivax* coinfection (see Table 2) and artemisinin-based combination therapy (ACT) for *P. falciparum* co-infection with a single dose of primaquine.

**Table 2:** Treatment policy for uncomplicated malaria

Parasite	Brazil	Colombia	Peru	Venezuela
*P. vivax*	CQ + PQ PQ at 0.50 mg/kg/day for 7 days (adopted 2006)	CQ + PQ PQ at 0.25 mg/kg/day for 14 days (adopted 1960s)	CQ + PQ PQ at 0.50 mg/kg for 7 days	CQ + PQ PQ at 0.25 mg/kg/day for 14 days (adopted 2004)
*P. falciparum*	AL + PQ(1d), Amazon^a^; AS + MQ + PQ(1d) (adopted 2012) non-Amazon areas PQ 0.75 mg/kg given on first day of ACT, changing soon to 0.25 mg/kg on first day of ACT	AL twice daily for 3 days; blisters 1.7 mg/kg artemether and 12 mg/kg lumefantrine per dose (adopted 2010)	AS + MQ (adopted 2001) + PQ 0.75 mg/kg single dose on last day of ACT (adopted 2015) [52]	AS + MQ + PQ (adopted 2004)

From [1]

*P. vivax: Plasmodium vivax*; *P. falciparum: Plasmodium falciparum*; AS: artesunate; AL: artemether–lumefantrine; CQ: chloroquine; MQ: mefloquine; PQ: primaquine; ACT: artemisinin-based combination therapy

^a^Expected to change soon to AS + MQ + PQ

According to the Amazon malaria initiative (AMI) annual report for 2015, in 2014 the percentage of *P. malariae* infections in Latin America was only 0.1% (Brazil, Colombia, Costa Rica, French Guiana, Guyana, Peru, and Venezuela) [5]. However, the real incidence of *P. malariae* in the region may be underestimated due to difficult microscopic differentiation from *P. vivax* as well as to low parasitaemia in coinfections; microscopists are not well trained in identifying this parasite due to its very low occurrence. In the early 1970s, *P. malariae* was identified in over 80% of all *Plasmodium* positive samples in an isolated Amerindian population in southeast Peru, with some cases of *P. malariae* coinfection with *P. vivax* [32]. Using molecular detection such as nested PCR to amplify a species-specific sequence of the 18S SSU rRNA gene in blood samples of 497 individuals living in an endemic region of the Brazilian Amazon basin, a report from 2004 found 11.9% (59 out of 497) of *P. malariae* cases while microscopic examination of the same samples showed only 1.2% (6 out of 497) [33]. A 2005 study from Suriname reported 12% *P. malariae* infections in the population [34]. A very recent report showed that in a region of the Colombian Amazon *P*. *malariae* infections were almost half (43.8%) of all symptomatic malaria cases tested by nested PCR [35]. The study revealed that 35.8% of the population had coinfections with two parasite species; the *P. vivax*/*P*. *malariae* combination occurred in 79.2% of coinfections. Because no *P. malariae* cases were reported in Colombia in 2015, the authors of this study suggest under-detection by the gold standard method of thick smear microscopy and poor detection regarding mixed infection and low parasitaemias. A very recent report from Colombia showed that *P. malariae* infections were not detected by microscopy, only by PCR; *P. malariae* infections were both single as well as mixed with *P. vivax* or *P. falciparum* [36]. Although *P. malariae* is present in small foci in communities of the Colombian Pacific coast [37] extensive cross sectional studies conducted recently in several endemic sites in this region using PCR have failed to identify this parasite species.

A very recent review of reports published between 1971 and 2016 of blood transfusion-transmitted malaria in the Americas showed that more than half of the malaria cases in which the *Plasmodium* species was identified were caused by *P. malariae* (58.4%), with 20.7 and 17.9% due to *P. vivax* and *P. falciparum* infection respectively [31]. Most cases occurred in Mexico (50.7%) and the USA (40.3%) followed by 6.6% in Brazil; from the cases in Brazil (n = 28) the *Plasmodium* species found was only identified in 3 cases, all caused by *P. malariae.*

### Possible zoonosis of two *Plasmodium* species in South America

Non-human primates (NHPs) are also infected with *Plasmodium* parasites, and thus they might constitute a natural reservoir for human malaria. The simian *Plasmodium brasilianum*, closely related to *P. malariae*, causes quartan fever in New World monkeys [26]. A very recent report shows natural human infection with *P. brasilianum* in the Venezuelan Amazon, affecting remote Yanomami indigenous communities [38]. The authors of this study suggest that, as opposed to previously thought, *P. malariae* and *P. brasilianum* are the same quartan malaria parasite species—an anthropozoonosis—that moves freely between NHPs and humans. Natural infections of monkeys in Panama, Venezuela, Colombia, Peru, Brazil, and French Guiana have been reported [39, 40]. *Plasmodium simium*, genetically related to *P. vivax* [41], is another parasite found in natural infections in Brazilian monkeys [42]. Whether these species can naturally infect humans, as has been suggested in a recently self-reported case in Rio de Janeiro Brazil for *P. simiun* [43], requires further investigation.

## Current surveillance: malaria case reports

In each of the four countries except for Brazil, the Ministry of Health (MoH) issues weekly epidemiological bulletins reporting on incidence of different diseases including malaria, for which data are shown as number of malaria cases, type of infection (parasite species), distribution per state and per district/municipality, distribution per age groups, risk maps, and often reporting number of detected imported cases (Table 3). These bulletins are available online. In Brazil, data gathering is handled by the MoH’s SIVEP-malaria (epidemiologic surveillance information system-malaria) in real-time, although delays have been reported depending on internet availability in the countryside [44]. The data gathered in Brazil is not publicly accessible but made available for online consultation at the municipality level (requires login) and local and regional authorities are instructed to routinely assess the data. In Colombia, a June 2014 malaria surveillance protocol states that uncomplicated malaria cases must be reported weekly, and complicated cases immediately, to SIVIGILA (public health surveillance and control system), and in Peru data is submitted to the national epidemiological network RENACE.

**Table 3:** Surveillance and epidemiological bulletin information

Surveillance	Brazil	Colombia	Peru	Venezuela
MoH malaria database	SIVEP malaria	SIVIGILA	RENACE	MoH (weekly epidemiological bulletin)^a^
Periodicity of reporting	Real-time	Weekly (non-complicated) Immediately (complicated cases)	Weekly	Weekly
Information in epidemiological bulletins	No epidemiological bulletins issued	*P. falciparum, P. vivax* or mixed infection Cases per region, municipality, age groups, ethnic groups, imported cases	*P. falciparum, P. vivax* or mixed infection Cases per department and districts and age groups	*P. falciparum, P. vivax, P. malariae* or mixed infection Cases per state, municipality and age groups

Weekly epidemiological bulletins can be downloaded from the MoH website for Colombia (http://www.ins.gov.co/boletin-epidemiologico/Paginas/default.aspx), Peru (http://www.dge.gob.pe/portal/index.php?option=com_content&view=article&id=347&Itemid=249), and Venezuela (http://www.mpps.gob.ve/)

^a^Interrupted after 44 week of 2014, with past (2015 and 2016) weekly bulletins made available online in 2017

## Anti-malarial treatment

In Latin America, chloroquine (CQ) is still widely used for vivax malaria treatment of asexual stages (blood schizonticidal). A total adult dose of 1500 mg/base is given over 3 days, in the countries reviewed here as a first day dose of 10 mg/kg followed by 2 days of 7.5 mg/kg each for a total dose of 25 mg/kg [45, 46]. Primaquine (PQ) for radical cure is also recommended as policy to be given along with CQ in a treatment regimen of either 14 consecutive days (Colombia and Venezuela) or 7 days (Brazil and Peru) for the same total dose (3.5 mg/kg) (Table 3) [47]. The shorter 7 days regimen is used in several developing countries as an attempt to reduce poor compliance associated with the longer 14 days regimen. However, in the Peruvian Amazon even this shorter regimen showed poor compliance (estimated at 62.2% adherence) between 2005 and 2007 among 1279 confirmed *P. vivax* patients [48]. Identified causes of poor compliance were perceptions that medication was bad, perceived allergies to medication or gastrointestinal side effects mainly associated with PQ (nausea, vomiting, stomach pain). Non-adherent patients abandoned treatment in the first 3 days, a period during which they feel sick, while during the second 4 days of only PQ they slowly improve, reducing the perceived benefits of more PQ days. In addition, patients had to return to the health centre to collect the remaining PQ tablets which may negatively affect adherence. In Brazil, adherence to vivax malaria treatment in the Amazon in patients also receiving primaquine for 7 days was 86.4% in a study on 282 patients who were visited and interviewed in their homes on the seventh day [49], whereas it ranged between 63.8% and 72.7 in another study involving vivax malaria 135 patients [50].

For falciparum malaria, ACT is used in the four countries, given over 3 days (Table 2). Artesunate (AS) plus mefloquine (MQ) is the regimen used in most countries. In Brazil, AS–MQ is used only in non-Amazon areas, whereas AL (artemether–lumefantrine) has been used in the Amazon since 2007 due to a fear of AS–MQ leading to artemisinin resistance because of the longer half-life of MQ. However, the recently reported lack of evidence regarding AS–MQ resistance in *P. falciparum* in the Brazilian Amazon reported recently [51] along with upcoming elimination scenarios have supported a decision to change back to AS–MQ therapy in this region, currently being discussed and expected to become a MoH recommendation.

A single dose of PQ as a gametocytocidal is added to ACT to prevent *P. falciparum* transmission in Brazil and Venezuela, with the recent addition in 2015 of Peru [52]. Colombia has not adopted this policy yet and does not use PQ as policy for *P. falciparum* infections. The *P. falciparum* PQ dose has been recently reduced by a new WHO recommendation from 0.75 mg/kg to a dose considered safe for individuals with glucose-6-phosphate dehydrogenase (G6PD) deficiency (0.25 mg/kg, see below) [53], however many countries still use 0.75 mg/kg. Brazil is expected to address this with an upcoming recommendation to use the single low dose of 0.25 mg/kg given the first day of treatment. Treatment of *P. malariae,* when diagnosed, consists of CQ without the need for PQ.

## Challenges for malaria elimination in the region

**Fig. 3:**
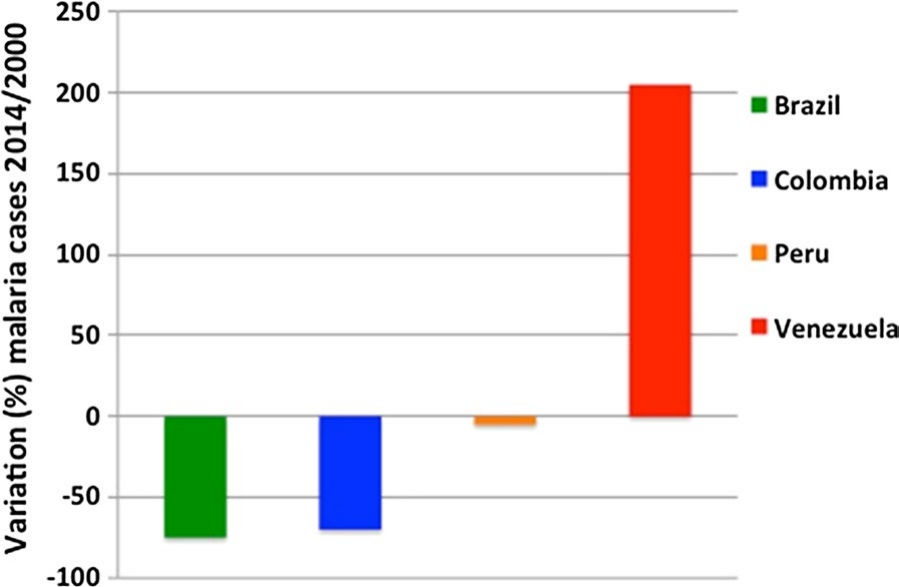
Change (%) in malaria cases in 2014 compared to 2000 baseline. 2014 reduction (%) in number of malaria cases relative to the baseline year 2000 in Brazil, Colombia, Brazil, Peru and Venezuela are shown Data from [2, 5]

During the 2000–2014 period, several countries in Latin America have achieved remarkable reductions in malaria incidence (Brazil over 75% decrease, Colombia 71.8% and Peru 5%, Fig. 3), with some entering pre-elimination and elimination phases. In 2014, Brazil reported its lowest number of malaria cases in 35 years and it has stepped up efforts towards *P. falciparum* elimination launching a National Elimination Plan aiming at reducing cases by 90% by the year 2030. In November 2015, the country’s NMCP was recognized as the 2015 Malaria Champion of the Americas [5].

Venezuela has in contrast shown a marked increase in malaria cases in the same time frame (Fig. 3), and Peru also experienced a steady increase since reaching its lowest point in 2011 [5]. As it is the case in Brazil, 95% of malaria cases in Peru occur in the Peruvian Amazon, in the Loreto region.

### Rapid increase in malaria incidence in Venezuela in recent years

Venezuela was the first WHO-certified country to eradicate malaria in its most populated areas back in 1961. However, the situation in this country is currently alarming. In 2012 Venezuela reported the highest incidence of malaria in its history until then, with 51,264 cases, surpassing the previous high of 5893 (12.6%) in 1990, most of them in Bolivar state (86.2% of the country cases), and three municipalities in “epidemic” and two in “alarm” status [54]. In Amazonas state, malaria increased and continued to occur mainly in children under 15 years (41.3%). Lack of anti-malarials and other malaria control measures in Venezuela have resulted in increasing number of cases in endemic and non-endemic regions recently, and this scenario affects neighbouring countries that receive imported cases of malaria, including *P. falciparum* infections, coming from Venezuela.

In a document prepared for and presented in September 2016 at the PAHO 68th session of the Americas regional committee for the malaria elimination project (action plan) 2016–2020, Venezuela was shown to have had a 205% increase in number of malaria cases in 2014 as compared to 2000 (Fig. 3). Except Haiti, which also showed an increase (much smaller than Venezuela’s), all other malaria endemic countries in the Americas experienced decreases in malaria incidence in the same time period.

Although no official publications were available since the government stopped publishing malaria epidemiological reports in late 2014, some have been later published online including all weekly reports for the years 2015 and 2016 which appeared in 2017. The re-emergence of malaria has resulted in a crisis that has been profiled in important international newspapers. A recent article in The New York Times indicated that currently cases show high morbidity and mortality due in part to the lack of available anti-malarial drugs such as CQ and PQ in affected regions, including gold mining areas in which mining is mostly illegal (see below) [55]. Men come to the mines looking for (usually temporary) jobs from nearby towns and cities due to higher mining wages, get infected with malaria while working at the mines, and bring it back home with them to the cities, resulting in whole families infected, as well as students and staff in schools near the mines. There is also now malaria in the capital city, Caracas [55].

Thirteen academic and public health Venezuelan institutions have come together to call attention to the current malaria epidemic in the country; with 146,670 cases registered until by August 27th for the year 2016, they have issued an alert that this is higher than the number of cases for the same time period in 2015 [56] and PAHO recently confirmed that the number of malaria cases for Venezuela in 2016 was 240,613, as published recently in 2017 on the weekly epidemiological bulletin corresponding to the end of 2016 (52nd week) by the Venezuelan MoH [7, 9]. Furthermore, malaria incidence in Venezuela may be greatly underestimated when based on recent reports due in part to administration of sub-therapeutic anti-malarial doses (3–7 days primaquine for vivax malaria) leading to recurrences that are not counted as new cases (Oscar Noya, pers. commun.). In the capital city Caracas, diagnosis of non-autochtonous cases at the “Centro para Estudios Sobre Malaria” used to be less than 60 cases per year until 2013; this number rose sharply to 377 cases between January and May of 2017, with approximately 80% *P. vivax* and 20% *P. falciparum* infections (Oscar Noya, pers. commun.).

The situation in Venezuela underscores the need for concerted malaria control efforts by Amazon countries in the region. About 78 and 81% of Brazil and Colombia malaria imported cases, respectively, are estimated to come from Venezuela [56].

### Border malaria and multinational efforts

Inter-country collaboration efforts at border areas may be needed, as demonstrated by the example of Ecuador and Peru eliminating malaria in recent years from adjacent coastal border regions [57]. Local public health practitioners from El Oro (Ecuador) and Tumbes (Peru) unofficially joined efforts in the mid-1990s for malaria control and eliminated malaria in the region in the following 20 years by strengthening surveillance and treatment strategies, sharing resources, conducting operational research to inform policy, and implementing novel interventions. El Oro has not had local malaria transmission since 2011 and Tumbes since 2012. These efforts contributed to detection of *P. falciparum* resistance to CQ (and subsequent implementation of appropriate alternative treatments even when these were not included in national policy). Active case detection strategies were implemented, and identified crucial strategies were strong community involvement, an extensive microscopy network and ongoing epidemiologic investigations at the local level. Regional partnerships in the region supported these efforts, including the Amazon network for the surveillance of anti-malarial drug resistance (RAVREDA), which along with the Amazon malaria initiative (AMI) have contributed substantially to malaria control efforts in South America, including the crucial policy change to ACT treatment of uncomplicated falciparum malaria of all these countries by 2006 [58].

An international initiative in the region supported by the Global Fund to fight AIDS, Tuberculosis and Malaria (GFATM) between 2005 and 2010 implemented interventions at the border areas of four countries (Colombia, Peru, Ecuador and Venezuela) that led to important reductions in malaria incidence. This project, called PAMAFRO (malaria control in the border areas of the Andean region), was part of an effort to work towards social integration of Andean countries particularly focusing on health and education. Malaria was chosen because of its international disease status of high priority in the region, affecting poor populations usually located in border areas. Among the malaria control strategies implemented there was critical community participation with local health workers involved and health education campaigns. During the period PAMAFRO activities were implemented, malaria declined in Peru by 95% for *P. falciparum* and 63% for *P. vivax* (reviewed in [59]).

### Urban malaria

Urban and peri-urban malaria, as opposed to the exclusively rural malaria in poor and isolated settings, is now a recognized phenomenon due in part to traveling but mostly to bigger population moves, especially from rural to urban and peri-urban areas. The origin of malaria cases found (diagnosed) in urban areas is not however clearly defined. In order to implement appropriate malaria control measures, it is important to determine whether these cases are being transmitted within the urban settings where they are detected or have been imported from rural settings. Urban malaria cases have been reported in Brazil, Colombia, and Venezuela [2].

A recent report on urban malaria across several Centres of Excellence for Malaria Research (ICEMR) including one each in Brazil and Colombia, and several others in Asia and Africa emphasizes that this determination requires epidemiological as well as entomological information about transmission [60]. They suggest that the strongest evidence for urban malaria transmission would be confirmed infection with no history of travel along with absence of *Anopheles* mosquitoes in the house, similar *Plasmodium* clones among humans and vectors in the urban area, as well as detection of gametocytes or sporozoites in infected mosquitoes [60].

In Brazil, the “free economic zone” economic model in the Amazon encouraged in the 1970s brought multinational companies to the region because of tax exemptions, resulting in uncontrolled waves of migration to the periphery of the city of Manaus from the interior of the Amazonas and other northern states [61]. There has been a gradual increase in malaria transmission in peri-urban areas afterwards, which has persisted to date. Malaria transmission in an urban/peri-urban riverside area in the Brazilian western Amazon (Rôndonia) was shown to be associated with a high prevalence of asymptomatic carriers in the population [62] and high anopheline vector densities [63].

In Peru, peri-urban malaria has also been reported in different cities in the Amazon (Iquitos [64]), three peri-urban squatter settlements in the northwest [65], and in the country’s capital Lima [66]. In Colombia, considerable migration has occurred as a result of armed conflict, illegal mining and agriculture, which have led to relocation to urban and peri-urban areas. A recent retrospective study based on 2008–2012 SIVIGILA official records showed that malaria cases suggested the probable presence of endemic, unstable and low-intensity malaria transmission in several urban and peri-urban municipalities located on the Pacific coast region and the eastern region [67]. However, it is unlikely that although malaria cases have increased in peri-urban areas, malaria in urban areas is autochthonous, as preliminary evidence has failed to confirm an urban origin for these cases as well as the presence of mosquitoes in these areas.

### Gold mining

Mobile populations such as miners, migrants, labourers are at high risk of malaria infection in the Americas. Malaria infection among miners in Brazil contribute about 6% of the country’s total cases, 3% in Colombia and 47% in Venezuela [2]. In the American continent, Brazil, Colombia, Venezuela, Suriname, French Guiana and Peru are countries with regions where significant gold extraction occurs which is associated with high malaria prevalence that presents mostly as asymptomatic cases [68–75].

Gold mining, often illegal in developing countries, affects ecosystems greatly as it starts with vast deforestation. Development of mining activities results in turn in highly mobile populations that migrate in search of jobs, usually young males, that work outdoors and are exposed to mosquito bites for long periods of time daily and sometimes live in camps with incomplete walls, allowing for an even wider exposure to mosquitoes. Deforestation and still water favour mosquito proliferation, especially *Anopheles darlingi*. Migration of previously unexposed to malaria populations into malaria endemic areas due to mining jobs has led to spikes in malaria cases in mining locations in all countries in this review. Furthermore, these areas usually lack good health care and disease prevention/control measures. The workers, upon return to their home villages, may introduce malaria parasites to new regions that could then be at risk of malaria depending on climate, activities and vector species present.

In Venezuela, gold mining has been associated with high malaria incidence in specific municipalities such as Sifontes where this activity is important, situated in the Bolivar state in the eastern region of the country [74]. The same phenomenon was observed in the North of Mato Grosso state in western Brazil [76–78] and in the southeastern Peruvian Amazon basin’s region “Madre de Dios” that held a large mining camp (Huepetuhe) that attracted migrants from the Andes, previously unexposed to malaria (reviewed in [70]). In Colombia, illegal gold mining has significantly increased during the last decade in areas with limited health care and lacking malaria control measures. During the 2010–2013 period, regions in which this activity was important, mostly on and near the Pacific and Atlantic coasts, contributed 89.3% (270,753 cases) of the national malaria incidence; of these, one-third (31.6%) of malaria cases were from mining areas [68]. The authors suggest that this may be an underestimation and malaria associated with mining activity and workers’ migration may be higher due to under-recording in these areas and high population mobility.

In French Guiana, recent data show that malaria affects many Brazilian gold miners working illegally in numerous mining sites [73]. Most of these illegal miners or *garimpeiros* work long hours and live in conditions of very poor hygiene and nutritional deficiencies, leading to poor health. Medical care is free in the French health centres, but the remoteness of the mines (sometimes 4 days by boat) and the fear of law enforcement hampers effective access to care by the miners. In Suriname, also with a considerable proportion of Brazilian miners (66%) [79] malaria transmission has decreased substantially due to the introduction of ACT; however gold miners remain vulnerable to malaria [75, 80].

### Malaria in pregnancy

Reports of malaria in pregnancy in South America are limited (reviewed in [81]). Malaria infections in pregnancy in low transmission areas may result in a higher risk of clinical disease with pronounced symptoms and malaria-related complications as compared to high transmission areas where pregnant females showed acquired immunity. Reports of malaria in pregnancy in the countries reviewed here highlight the importance of *P. vivax* infections in pregnancy.

There are a few recent reports on malaria in pregnancy from malaria endemic areas in Colombia that underscore the need for malaria diagnosis (preferably with a molecular method) and infection control as part of antenatal care in these areas. Two studies showed that malaria in pregnancy in these regions may be under diagnosed when evaluated by microscopy instead of PCR (some cases are asymptomatic). In a northwest Colombia area bordering Panama, the frequency of malaria in pregnancy for 2004–2007 detected as maternal blood infection by microscopy was 13% of women; however when assessed by PCR this rose to 32% [82]. Another study on 129 pregnant women in northwest Colombia from 2008 to 2011 found that the prevalence of gestational malaria (microscopy/PCR) was 9.1/14.0% (65% cases caused by *P. vivax*) and 3.3/16.5% for placental malaria (mostly caused by *P. falciparum*) [83].

In Colombia, malaria in pregnancy can be caused both by *P. falciparum* and *P. vivax* infections, and although most cases that progress into severe malaria are due to *P. falciparum*, *P. vivax* can also result in severe malaria. Although Colombia uses PQ for vivax malaria, it is contraindicated in pregnancy and pregnant women may, therefore, have recurrent infection due to relapses of the dormant liver stages (hypnozoites) which can only be eliminated with PQ. A report on a retrospective study from pregnancies in 2005–2006 in the Colombian northwest found severe malaria in pregnancy with hepatic dysfunction and spontaneous bleedings as the most frequent complications present in >70% of severe cases [84]. This study showed cases of both *P. falciparum* and *P. vivax* infections in pregnancy, with an almost 20% of *P. falciparum* infections being afebrile (asymptomatic), at risk of being undetected. Another report from three malaria-endemic areas of Colombia of a descriptive study of 34 malaria in pregnancy cases from 2011 to 2013 included three (8.8%) severe *P. falciparum* malaria cases with moderate anaemia (mean Hb 8.7 ± 0.1 g/dL) and an additional two severe malaria cases in women who developed a second malaria episode during pregnancy (and *P. falciparum,* one *P. vivax*) [85]. Of the first episodes, two women developed prostration and required hospitalization, and one had severe thrombocytopaenia; and of the second episodes (both in 15 year-old females) the *P. falciparum* infection presented hepatic dysfunction with hyperbilirubinaemia and clinical jaundice, while the *P. vivax* case showed severe anaemia (Hb 4.4 g/dL) with general pallor. The second malaria episode during pregnancy in six women was due to the same parasite species as the first infection (one *P. falciparum,* five *P. vivax*) but with lower parasitaemia in the *P. vivax* infections (median 6759 and 3030 parasites/µL, respectively) and lower Hb levels. The patients had received treatment without PQ for *P. vivax* as it is contraindicated in pregnancy.

In Brazil, where PQ is also not prescribed to pregnant women (who receive anti-relapse prophylaxis consisting of CQ 7.5 mg/kg weekly throughout pregnancy) studies have shown that malaria in pregnancy has a higher prevalence than malaria in non-pregnant women of similar age in endemic regions, and most of it (about 80%) is due to *P. vivax*. Out of the 13,308 malaria cases reported in Manaus from 2003 to 2006 among women aged 10–49 years, 6.1% were in pregnant women and 80% of these were due to *P. vivax* [86]. In 2007–2008, malaria cases diagnosed in pregnant women comprised 6.7% of fertile-age women in three municipalities located in the Amazon Region, 80% of which were due to *P. vivax* infection [87]. A recent report from a low endemicity (non-Amazon) southeastern area found autochthonous cases of malaria in pregnancy of women infected by *P. vivax* and *P. malariae* (1.6% by microscopy, 5.6% by PCR) with most cases from a rural area near the forest [88]. Brazil has recently introduced, in malaria endemic areas, a malaria result field in the prenatal card to ensure that pregnant women in endemic areas are tested for malaria [12].

In contrast, a retrospective analysis of pregnant women living in the Amazon region of Iquitos in 2004–2005, Peru, showed a lower prevalence of clinical malaria in pregnant women, although the authors suggest this may be due to passive case detection and surveillance bias, and community-based data including active case detection suggested that this population may have a higher frequency of asymptomatic infections and higher incidence of *P. falciparum* compared with non-pregnant male and female counterparts in the same age groups [89]. The use of only microscopy for diagnosis and not PCR in this study, as shown by studies in Colombia (see above) may have led to under diagnosing of (mostly asymptomatic) malaria in pregnancy cases. Indeed, a report on 193 malaria in pregnancy cases in the same region for 2004 showed a 1.0 and 6.6% positive cased as detected by microscopy and PCR respectively and demonstrated significant subclinical malarial infection in this population associated with increased presence of monocytes in the placenta [90]. The importance of *P. vivax* infection in severe malaria in pregnancy in Peru is highlighted in a case report that occurred in 2007: a 19 year-old pregnant woman with a history of travel was admitted to the hospital and later diagnosed with severe *P. vivax* infection when transferred to the intensive care unit (ICU) with multiple organ failure and stillbirth, leading to death [91].

In the southwestern Bolivar state of Venezuela (which contributes most of the malaria cases in the country), a descriptive malaria epidemiological study including pregnant women conducted in 2005–2006 revealed an incidence of malaria in pregnancy of 27.4%: 87% *P. vivax* with almost three quarters presenting symptoms [92]. In this study, a higher proportion of abortions occurred among *P. vivax* infected mothers (3/5); and one case of *P. vivax* placental malaria (0.8%) was registered.

### Drug resistance

Anti-malarial drugs, especially when given as monotherapy in the past for a period of time, have resulted in drug resistance due to mutations arising in target parasites such as *P. falciparum* or *P. vivax.* In the early 1960s, CQ resistance in *P. falciparum* appeared in migrants working in endemic malaria areas along the Thailand–Cambodia and Colombia and Venezuela border areas and later spread to Africa in the late 1970s probably imported by labourers from Southeast Asia [93–95]. Currently, WHO recommends ACT as first-line treatment for uncomplicated falciparum malaria, and all countries in this review follow this recommendation as policy, adopted in the 2000s. Resistance to artemisinin in *P. falciparum* has arisen in Southeast Asia [96] with subsequent identification of a molecular marker in the propeller domain of the *kelch13* (*k13*) gene [96–98], however ACT has been effective so far elsewhere including in the South American countries reviewed here [51], although surveillance for resistance markers is recommended in neighbouring regions such as Guyana and in mining areas in the region where artemisinins are available for sale and self-treatment is common [99].

A retrospective survey using medical records from 1992 to 2008 in Manaus, Brazil, showed that parasite clearance detected by microscopy in patients with *P. falciparum* infection treated with ACT (N = 1554) was reported by day 4 in 1528 (98.3%) patients, whereas there was delayed clearance (between days 4 and 7) in the remaining 26 patients (1.7%) (unpublished observations). Although a recent global survey of ACT resistant parasites which included South American samples from the Peruvian and Brazilian Amazon as well as from the Colombian coast indicated that all parasite isolates analysed from this region lacked *k13* mutations associated with resistance [100], these results should be interpreted with caution. A very recent report from Cambodia has identified artemisinin-resistant *P. falciparum* isolates without *k13* mutations, indicating that there may be additional genes that when mutated contribute to artemisinin resistance in these parasites [101].

In the Americas, CQ is still used widely for vivax malaria. Although there are reports of CQ resistance in the Brazilian Amazon [102, 103] where the majority of malaria cases and especially hospitalizations are due to this type of malaria, CQ is believed to remain quite effective. Although not conclusive, one of these studies suggests a possible association between anaemia and CQ resistance [103]. Regular drug resistance surveillance is suggested, although reliable assays based on genotyping are not yet available due to the lack of a validated molecular marker associated with CQ resistance in *P. vivax.*

### Submicroscopic asymptomatic infections

In the Americas, malaria diagnosis by microscopy analysis of thick and thin blood smears remains the standard method, which requires trained microscopists able to identify different stages of parasite morphology and distinguish between *P. falciparum* and *P. vivax* infections. Rapid diagnostic tests (RDTs) are being encouraged to some extent in Brazil and Colombia especially in remote areas with no easy access to microscopy facilities (Table 4). They detect specific parasite antigens from a finger prick blood samples and the availability of “combo” tests allows correct diagnosis or mono or mixed infections at once. Brazil’s distribution and use of RDTs has increased from 1486 tests in 2011 to 14,655 in 2015, especially in areas without good microscopy capability [12].

**Table 4:** Malaria diagnosis

	Brazil	Colombia	Peru	Venezuela
Diagnostic methods	Mostly microscopy (98%) [12] RDT: *P. falciparum* and all species combo, used in remote areas or those without easy access to microscopy (e.g. indigenous areas, legal mining camps) but not for surveys or treatment follow-up	Mostly microscopy RDT: *P. falciparum* and *P. vivax* combo	Microscopy	Microscopy

*RDT* rapid diagnostic test

Most *P. falciparum*-specific RDTs detect the *P. falciparum* histidine-rich protein 2 (HRP2). However, reports from Peru starting in 2010 revealed the existence of *P. falciparum* parasites containing deletions of the *pfhrp2* gene in the Peruvian Amazon region [104, 105] resulting in false negative HRP2-based RDT results [106]. These reports showed the presence of *P. falciparum* isolates with *pfhrp3* deletions as well, and some parasites containing both *hrp2* and *hrp3* gene deletions. In areas of low malaria transmission such as the four countries reviewed here, *P. falciparum* parasite densities in infected individuals are usually low, further increasing the probability of a false negative result. An alternative *P. falciparum* test in regions known to have *pfhrp2* deletions is one based on detection of *P. falciparum* lactate dehydrogenase (LDH). This test performs well in the areas reported to be affected by *pfhrp2* deletions in Peru [106], although available LDH RDTs show lower sensitivity at low parasite densities. To detect possible new areas with *pfhrp2* deletions, WHO has recently recommended to further analyse *P. falciparum* isolates from infections showing a discordance between HRP2-RDT and microscopy results (≥10–15% higher positivity microscopy rates) by PCR first to confirm the presence of *P. falciparum* and then proceed to confirm a possible *pfhrp2* gene deletion by PCR and antigen analysis [107].

An increasing number of studies are being published that performed molecular diagnosis of malaria by detection of *Plasmodium*—specific nucleic acids based on their amplification by ultrasensitive techniques, including using a high-volume sample quantitative polymerase chain reaction (qPCR) method for the detection of low-density parasitaemias (>20 parasites/mL) [108]. In Asia and Latin America, both low transmission malaria settings, there is now strong evidence showing that a significant proportion of positive cases detected by molecular methods is missed by microscopic examination of blood smear samples, as well as by RDTs [109–113] (Table 3). High PCR sensitivity over microscopy and RDT diagnosis was confirmed by studies from Canada that assessed the prevalence of malaria in asymptomatic refugees [114, 115].

The PCR-positive cases that are microscopy- or RDT-negative are usually asymptomatic infections that go undetected also due to low parasitaemias in subjects that may be screened by non-molecular methods as part of malaria surveys in endemic regions. The parasites in these infections constitute a reservoir that contains infective gametocytes that can indeed be transmitted to mosquitoes, as shown by a recent study from Colombia with natural or experimentally-induced *P. vivax* infections [116]. Asymptomatic *P. vivax* carriers from the Brazilian Amazon were able to infect mosquitoes at a rate of about 30% ([117], unpublished observations). Asymptomatic infections in three neighbouring Brazilian states (Mato Grosso, Amazonas and Rondônia) have been reported, as well as among indigenous communities in the Colombian, Peruvian, and Venezuelan parts of the Amazon basin adjacent to Brazil (reviewed in [118]). These asymptomatic infections seem to be more common in older people (suggesting an association with acquired immunity) and those with longer time residing in the area. Recent PCR-based field studies have revealed that in Venezuela the number of asymptomatic infections may be fourfold higher than in symptomatic individuals with a great predominance of *P. vivax* infections (Oscar Noya, pers. commun.). Ongoing studies in Amerindian communities indicate that asymptomatic infection prevalence may be even higher, also with a vast majority of *P. vivax* infections; upcoming studies will similarly address asymptomatic infections in gold mining populations (Oscar Noya, pers. commun.).

A molecular method that has been evaluated as an alternative to the more expensive PCR technique is the loop mediated isothermal DNA amplification (mLAMP) [119], a faster and more cost-effective method for amplification of parasite nucleic acids. Available LAMP kits would be more suitable for use in field settings as they require considerable less training and minimal equipment as compared to PCR. A mLAMP kit with sensitivity as low as detection of 1 parasite/µl of blood in less than 1 h was recently tested in individuals with asymptomatic malaria in remote endemic areas of Colombia where *P. vivax* predominates [120]. The study found comparable sensitivity and specificity for mLAMP and RT-PCR for detection of both *P. falciparum* and *P. vivax* infections resulting in increased detection of asymptomatic malaria infections. A recent report from Colombia showed single infections only identified by PCR (negative by microscopy) and mixed infections were revealed by PCR analysis that had been regarded as single infections, including those of *P. vivax* or *P. falciparum* with *P. malariae* (see above); the later parasite was only detected by PCR [36].

In a cross-sectional study conducted in 2012 in the Manaus area [121], 4.3% of all participants were infected with *P. vivax* as determined by qPCR, 2.4-fold higher than prevalence detected by microscopy (1.8%). Furthermore, from all *P. vivax* infected individuals, about half (46.8%) were positive for gametocytes as detected by qRT-PCR and 82.7% did not report a concurrent febrile illness (asymptomatic). In the same study, *P. falciparum* infections were rare (0.8% by qPCR) of which 73.3% were asymptomatic.

### Vector control

There is a great diversity of *Anopheles* mosquito species In the Americas, with several acting as malaria vectors. Their ecology and biting behaviour are not well studied, and show regional variation. The major malaria mosquito vector in the countries reviewed here is *Anopheles darlingi* [122], a very efficient anthropophilic species that can bite indoors and transmit both *P. falciparum* and *P. vivax*. It is the main malaria vector species in the western Brazilian Amazon [123]. In Colombia, several *Anopheles* species have been reported to be involved in malaria transmission including *An. darlingi* along with *An. nuneztovari* and *An. albimanus* [124].

In Latin America, including the four countries reviewed here, vector control methods have been implemented in endemic areas, mostly the use of long-lasting insecticide treated nets and indoor residual spraying (IRS). However, there is a knowledge gap on the impact that these measures have on suppressing mosquito vector populations and parasite transmission.

All four countries recommend the use of insecticide-treated nets (ITNs) and long-lasting insecticidal nets (LLINs), which are distributed free of charge to all age groups (Table 5). IRS is also recommended in national policies, with no DDT used in any of the four countries. Larval control is only recommended in Colombia and Venezuela ([1], Table 5). In Brazil, there is some larval control with environmental management based on cleaning of selected breading sites. In western Colombia, a recent study on larval habitats of malaria vector species in endemic areas showed that important habitats were man-made water bodies: fishponds for fish rearing, water wells for home water supply, and excavation sites [125]. Authors suggest that these habitats should be targeted for larval control in a safe manner to avoid damage to fish in ponds or to humans who drink water from the wells.

**Table 5:** Vector control policies

	Brazil	Colombia	Peru	Venezuela
ITNs/LLINs	Distributed free of charge to all age groups since 2007	Distributed free of charge to all age groups since 2005	Distributed free of charge to all age groups	Distributed free of charge to all age groups since 2005
IRS	Recommended	Recommended	Recommended	Recommended
Larval control	No	Recommended	No	Recommended

From [1]

*ITN* insecticide-treated nets, *LLINs* long-lasting insecticidal nets, *IRS* indoor residual spraying

### G6PD deficiency and primaquine use

Glucose-6-phosphate dehydrogenase (G6PD) deficiency (G6PDd) is a widespread enzyme deficiency that affects approximately 400 million people worldwide (reviewed in [126–129]). G6PD is a critical protector from oxidative stress. In red blood cells, G6PD activity decreases exponentially with cells’ age (half-life ~50 days). The gene encoding G6PD is highly polymorphic, resulting in about 400 variants, most of them consisting of amino acid substitutions caused by specific mutations [130, 131]. Depending on the prevalence of G6PDd in malaria endemic regions, the use of PQ as indicated in Tables 2 and 3 is a concern, as this 8-aminoquinoline drug can acute haemolytic anaemia (AHA) of variable severity in G6PDd individuals in a dose-dependent manner.

Two recent reviews of G6PDd provide some prevalence estimates worldwide and for Latin America (LA) ([132, 133] and both clearly show that there is limited data from the Americas. The first one (predictive model based on published studies) predicted an overall allele frequency of 8.0%, lowest in the Americas and highest in tropical Africa, while the second analysis of published literature showed a great heterogeneity of G6PDd prevalence geographically in the Americas, even within countries, an example best illustrated by the case of Brazil (0–8% range with regions of 0–2, 2–4, 4–6 and 6–8%) [133].

The most frequent G6PD deficiency variant in Africa, known as A−, seems to be also the most predominant in South America, although as it occurs in the malaria as well as other tropical disease fields, reports from South America are not abundant. In contrast, in Asia several different variants have been described (and new ones emerging frequently from genotyping studies) which sometimes result in extreme degrees of deficiency. G6PD A− is considered to be less severe in comparison. The G6PD A− variant is believed to have came to the Americas with West African populations during the transatlantic slave trade. The G6PD A-202A mutation is the variant most broadly distributed, identified in 81.1% of the G6PDd individuals surveyed in the Americas [133]. Reports from Colombia, Brazil and Venezuela show that this variant is the predominant genotype found among G6PDd samples [134–137]. Peru, on the other hand, has a very low prevalence of G6PDd (Table 6).

**Table 6:** G6PDd prevalence and primaquine policy

Country	Estimated G6PDd for common variants (mostly A −)	PQ use	PQ as DOT?	G6PD testing required for PQ administration
Brazil	>3–7% [132] 0–12.9% [133]	*P. falciparum* and *P. vivax*	No	No-currently under consideration and expected to be recommended when possible with follow-up of possible PQ-induced haemolysis G6PDd cases to be treated with PQ at 0.75 mg/kg/week for 8 weeks
Colombia	>3–7% [132] 1.4–15.4% [133] 6.56% [135]	*P. vivax*	No	No
Peru	>0–1% [132] 0–0.7% [133]	*P. falciparum* (since 2015) and *P. vivax*	Yes	No
Venezuela	>7–10% [132] 0–3.5% [133] 3.6% [134]	*P. falciparum* and *P. vivax*	No	No

PQ and G6PD testing policies taken from [1]

*P. vivax: Plasmodium vivax*; *P. falciparum: Plasmodium falciparum*; DOT: directly observed treatment; PQ: primaquine. PQ is contraindicated in infants and pregnant and breastfeeding women

A recent study from four malaria endemic areas in Colombia found a 6.56% of G6PDd overall (N = 426) including both intermediate and severe deficiency [135]. Previous reports of G6PDd prevalence in Colombia varied (as discussed for Brazil above) depending on the region and/or specific population assessed: higher on the coasts (12% in Buenaventura (Pacific coast) for 242 individuals [138], 14.8% in 508 individuals in Turbo (Atlantic coast) [139]), lower in the capital Bogotá (3.1% in a mestizo population) [140]. A report from 1968 that used a G6PD activity test based on discoloration of brilliant cresyl blue to survey 500 Colombian males in different sub groups based on race classification at the time found 1.2–2.5% G6PDd in white and urban groups, 13.5 and 22.2% respectively in two different groups of African descent, and interestingly, no G6PDd in indigenous people (n = 45) [141]. This latter observation was confirmed in 3739 Brazilian indigenous individuals tested for whom no G6PD variants were found [142]. Even though the prevalence of G6PDd in Colombia is considered high, there are no available reports of haemolytic crises in malaria endemic communities after PQ treatment. Ongoing studies are addressing this possible knowledge gap by assessing potential development of haemolysis in these populations. This information is needed to help design appropriate malaria elimination strategies that require PQ administration.

Implementation of malaria pre-elimination and elimination programmes will rely on the use of PQ to prevent *P. falciparum* transmission and *P. vivax* relapses, therefore knowledge of local G6PDd prevalence should be taken into account. Although none of the malaria endemic countries in the Americas has currently as policy a requirement for G6PD testing prior to PQ administration, Brazil is currently considering to introduce a recommendation to test, whenever possible, for a *P. vivax* diagnosis before PQ treatment. An alternative safer treatment for positive G6PDd cases identified would be PQ at 0.75 mg/kg/week for 8 weeks. The lack of a good qualitative rapid G6PD activity test to be used in the field is a challenge. The chromatographic CareStart™ test has been recently tested in the Brazilian Amazon in 674 individuals (320 of whom where infected with *P. vivax*) and shown to have low sensitivity in detecting mild and intermediate G6PDd, however its high sensitivity in detecting severe deficiency makes it a good candidate as a RDT for G6PDd [136]. A recent study comparing two G6PD activity tests in the same region showed that CareStart™ was cost-effective in diagnosing G6PDd and avoiding hospitalization [143].

From the four South American countries reviewed here, only policies in Brazil and Venezuela recommend the use of PQ as gametocytocidal treatment of *P. falciparum* infections (Table 3). All countries have radical treatment of *P. vivax* with PQ as policy, recommended along with CQ, at 0.25 mg/kg for 14 days in Colombia and Venezuela, whereas in Brazil and Peru the same total PQ dose is given over a shorter time (0.5 mg/kg/day for 7 days) (Table 2). Only in Peru the policy recommends PQ administration as directly observed treatment (DOT) (Table 4).

The incidence of PQ-induced haemolysis in G6PDd individuals in the Brazilian Amazon area has been underestimated [12], as evidenced also by reports from a case series [144] and an autopsy series of deaths due to vivax malaria [22] both from Manaus. From a prospective study of 316 *P. vivax*-related admissions in the same city [21], 45 patients presented PQ-induced haemolysis, with 17 requiring blood transfusion and 4 requiring dialysis due to acute renal failure (unpublished observation). Although there is currently a decrease in the number of hospitalizations in Manaus due to malaria, there is a simultaneous increase in hospitalisations due to PQ-induced haemolysis (unpublished observation).

## Conclusions

The situation in Venezuela is critical; national and regional efforts are needed to contain malaria spread especially in mining areas, focused on diagnosis, vector control and stocking of appropriate antimalarial treatment drugs. The region would benefit from a greater political commitment towards malaria elimination to overcome remaining challenges through collaboration between health authorities, academia and communities. A great model of regional cooperation focused on a common malaria elimination goal is the Asia Pacific Malaria Elimination Network (APMEN) Vivax Working Group, a collaboration of 18 countries that has worked successfully in a joint effort to build knowledge, tools, expertise, evidence and regional consensus based on identified knowledge gaps to effectively change practice [145]. In South America, RAVREDA/AMI and PAMAFRO have contributed in the past to malaria control and elimination strategies implementation; renewed regional networking initiatives in this direction are needed.

Hard to reach isolated populations (i.e. indigenous, riverine, miners) for whom traditional health system practices are largely unavailable remain a considerable challenge for case detection. On the other hand, urban malaria requires further investigation into possible transmission occurring within cities where cases are reported.

The data presented here supports the following recommendations:

- Implementation of a field-compatible molecular test for malaria diagnosis in rural areas;
- Testing that can detect *P. malariae* infections in addition to *P. falciparum* and/or *P. vivax* in areas where this parasite has been reported;
- Malaria in pregnancy, an important population for malaria control and elimination programmes, calls for malaria diagnosis and appropriate treatment for positive cases as part of regular antenatal care visits, especially in remote malaria endemic areas.
- Implementation of G6PDd field-compatible diagnostic tests to guide PQ administration, especially for *P. vivax* infection which causes the majority of malaria cases in the endemic areas reviewed here with.
- An alternative (8 weeks) weekly PQ regimen for vivax malaria for G6PDd individuals, preferably as DOT and/or with specific instructions to patients regarding alarming haemolysis signs such as dark urine.
- Addition of SLD PQ for *P. falciparum* infections in Colombia, especially on the Pacific coast where there is a higher incidence of falciparum malaria.

## Abbreviations

ACT: artemisinin-based combination therapy
AL: artemether–lumefantrine
AMI: Amazon malaria initiative
AS: artesunate
CQ: chloroquine
DOT: directly observed treatment
G6PD: glucose-6-phosphate dehydrogenase
G6PDd: G6PD deficiency
PAHO: Pan American Health Organisation
PAMAFRO: malaria control in the border areas of the Andean region
PQ: primaquine
RAVREDA: Amazon network for the surveillance of antimalarial drug resistance
RDT: rapid diagnostic test
SLD: single low dose
WHO: World Health Organization
